# Proceedings of the Association of Representatives of American Medical Colleges

**Published:** 1876-06

**Authors:** J. B. Biddle, Leartus Connor

**Affiliations:** President; Louisville, Ky.; Sec’y.; Louisville, Ky.


### Miscellaneous.

### Proceedings of the Association of the Representatives of American Medical Colleges, held at Philadelphia, June 2d and 3d, 1876.

A convention of representatives of numerous medical colleges of the United States was held in the hall of the Jefferson Medical College, of Philadelphia, June 2d and 3d, 1876, in pursuance of the following call:

LOUISVILLE, KY., May 15, 1876.

Following a general correspondence with the various medical colleges of the United States, the undersigned issued this call for a convention, to be held in Philadelphia, on Friday, June 2, 1876, four days in advance of the American Medical Association. The object of the convention is to consider all matters relating to reform in medical college work.

That decided results may be reached, the faculty of each college is requested to send one or more delegates, clothed with *plenary* powers to determine final action on every question.

Should any college find it impracticable to send a representative, it is hoped that it will set forth fully by letter to the convention the views it may hold touching the suppression of existing evils and methods of practical improvement.

Officers of the following colleges have informally signified their hearty approval of the movement:

Jefferson Medical College, College of Physicians and Surgeons, N. Y., Bellevue Hospital Medical College, Ohio Medical College, Miami Medical College, Rush Medical College, Detroit Medical College, Louisville Hospital Medical College, Keokuk Medical College, Cleveland Medical College, Starling Medical College, Medical Department of Georgetown College, Medical Department of Columbia University, Long Island College Hospital, Medical Department of Syracuse University, Evansville Medical College, Indiana Medical College, Medical Department of University of Nashville, Atlanta Medical College, Mobile Medical College, Savannah Medical College, Augusta Medical College.

The convention will be called to order in the hall of the Jefferson Medical College at 11 o'clock A. M. on the day named above.

J. B. BIDDLE, M. D., Jefferson Medical College.
WM. H. MUSSEY, M. D., Miami Medical College.
JOHN. T. HODGEN, M. D., St. Louis College.
J. ADAMS ALLEN, M. D., Rush Medical College.
W. T. BRIGGS, M. D., Med. Dep't University of Nashville.
J. M. BODINE, M. D., Med. Dep't University of Louisville.

At the hour named the following representatives assembled:

Jefferson Medical College—Prof. J. B. Biddle and Prof. S. D. Gross.

Medical Department University of Pennsylvania—Prof. R. E. Rogers.

College Physicians and Surgeons of New York—Prof. Edward Curtis.

Medical Department University of Louisville—Prof. L. P. Yandell, Jr., and Prof. J. M. Bodine.

Hospital College of Medicine of Louisville—Prof. J. A. Larabee and Prof. T. C. Wilson.

Long Island Hospital Medical College—Prof. J. H. Raymond.

Medical Department University of Iowa—Prof. E. Clapp.

College of Physicians and Surgeons Syracuse University—Prof. H. B. Wilbur and Prof. Van Dyne.

Chicago Medical College—Prof. L. Curtis.

Medical Department University of Georgia—Prof. E. Geddings.

Indiana Medical College—Prof. T. B. Harvey and Prof. L. D. Waterman.

Medical Department University of Wooster—Prof. W. J. Scott.

Cleveland Medical College—Prof. J. H. Bennett and Prof. Heims.

Detroit Medical College—Prof. E. W. Jenks and Prof. L. Connor.

Starling Medical College—Prof. S. Loving.

Medical Department University of Vermont—Prof. H. D. Holton.

St. Louis Medical College—Prof. J. L. B. Alleyne.

Atlanta Medical College—Prof. W. F. Westmoreland.

Medical Department University of Nashville—Prof. W. T. Briggs.

Medical Department Vanderbilt University—Prof. T. A. Atchison.

Missouri Medical College—Prof. A. P. Lankford.

Keokuk College Physicians and Surgeons—Prof. J. J. M. Angier.

Columbus Medical College—Prof. J. F. Baldwin.

On motion of Prof. Yandell, Prof. J. B. Biddle was elected President of the convention, and on motion of Prof. Bennett, Prof. Leartus Connor was elected Secretary.

On motion of Prof. E. Curtis, it was

*Resolved,* That the action of the convention shall not be considered binding upon the colleges represented unless endorsed by their respective faculties.

On motion of Prof. Gross, it was

*Resolved,* That a committee be appointed to submit business for the consideration of the convention, to report at the afternoon session.

The chair appointed as this committee, Profs. Bodine, Gross, Geddings, Holton and Scott.

The convention adjourned until 4 P. M.

Pursuant to adjournment, the convention reassembled at 4 P. M., the President in the chair.

The minutes of the last meeting were read and approved.

Prof. Bodine, from the committee to prepare business for the convention, reported the following questions for its consideration:

1. Shall the beneficiary system, with its present abuses, be condemned or endorsed?

After discussion, on motion of Prof. E. Curtis, the following preamble and resolutions were adopted with reference to question first:

*Whereas,* The practice of reducing or remitting in individual cases the established fees of a college has the objectionable feature of discriminating between students who may be equally deserving, and opening the door to possible gross abuses; therefore

*Resolved, first,* That this convention regards the above privilege as one to be depreciated in general, and, if put into practice at all, to be exercised both rarely and reluctantly, and only in unusual circumstances, and after unsolicited application by proven deserving candidates.

*Resolved, second,* That anything like a wholesale system of such reduction or remission of established fees, or any open solicitation of recipients of such favors be regarded as in the highest degree improper, and that any college indulging in such practices deserves to forfeit its place on the *ad eundem* list of medical colleges.

Question 2. Shall two consecutive courses of lectures in one year entitle students to become candidates for graduation?

On motion of Prof. E. Curtis, it was

*Resolved,* That it is the opinion of this convention that no two consecutive sets of lecture tickets shall be regarded as fulfilling the usual pre-requisites of instruction for graduation, where the time between the beginning of the first course and the end of the second is less than fifteen months.

Question 3. Shall any faculty under any circumstances issue a diploma not bearing the graduate's name.

On motion of Prof. Waterman, it was

*Resolved,* That no medical faculty should issue a diploma not bearing the graduate's name.

It was ordered that the meetings of the convention shall be at 10 A. M. and 4 P. M. On motion, the convention adjourned.

The convention reassembled on Saturday, June 3d, at 10 A. M., the President in the chair.

The minutes of the previous meeting were read and approved.

On motion of Prof. L. P. Yandell, Jr., the regular order of business was suspended, and communications were read from the faculties of the following medical colleges: Louisville Medical College, Kentucky School of Medicine, Evansville Medical College, Rush Medical College, Medical Department University Louisiana,

Medical School of Harvard University, Savannah Medical College, Cincinnati College of Medicine and Surgery, Medical College of State of South Carolina.

On motion of Prof. Atchison, these communications were placed on file.

Question 4. Shall this convention resolve itself into a permanent organization?

On motion of Prof. Atchison, it was

*Resolved*, That the question be referred to a committee of five, to report at the afternoon session.

The chair appointed as this committee Profs. Atchison, L. Curtis, E. Curtis, Yandell and Scott.

On motion of Prof. Rogers, the President and Secretary of the convention and Prof. Atchison were appointed a committee on publication.

Question 5. Is there any reason why the customary diploma fee shall be abolished?

On motion of Prof. Rogers, it was

*Resolved*, That it is the sense of the convention that the diploma fee should not be abolished.

Question 6. Is it advisable to adopt a graded course of study?

On motion of Prof. Bodine, the following preamble and resolution were adopted in reference to this question:

*Whereas*, A knowledge of the elementary branches of medicine should precede a study of the practical branches.

*Resolved*, That, in the hope of inducing students to prolong and systematize their studies, this convention recommends to all medical colleges to offer to students the option of three courses of lectures, after a plan similar to the following: Students who have attended two full courses of lectures on anatomy, chemistry, materia medica and physiology, may be examined upon any of these subjects at the end of their second course. During their third course such students may devote themselves to the lectures upon the theory and practice of medicine, surgery, obstetrics and diseases of women and children, upon which subjects only they shall be examined at the final examination for the degree of M. D.—their standing, however, to be determined by the results of both examinations.

On motion, adjourned till 4 P. M.

The convention re-assembled at four P. M., the President in the chair. The minutes of the last meeting were read and approved.

Prof. Atchison, from the committee to whom the subject of permanent organization was referred, reported the following resolutions:

*Resolved*, 1. That this *convention* now proceed to form a Provisional Association of American Medical Colleges, under its present officers.

*Resolved*, 2. That when the Association adjourns, it shall adjourn to meet at the call of its President.

*Resolved*, 3. That the various medical colleges be invited to take into consideration the project of forming, at the next meeting of this Provisional Association, a permanent Association of American Medical Colleges.

*Resolved*, 4. That for the furtherance of this object, a committee of three be appointed at this meeting to confer by letter with the various colleges, and invite their views on the proper object and plan of such proposed organization; and upon the receipt of the same, to draft a constitution and by-laws for a permanent Association, to be submitted at the next meeting of this Association.

*Resolved*, 5. That the advisory resolutions upon matters of college policy passed by this convention be printed and forwarded to all medical colleges in the United States for their consideration.

The chair appointed as committee to carry out the foregoing resolutions Prof. T. A. Atchison, Prof. Edward Curtis and Prof. L. P. Yandell, Jr.

These resolutions were adopted, and the convention resolved itself into the Provisional Association of American Colleges.

Question 7. Is it proper for a regular college to have any kind of alliance with homeopathy?

On motion of Prof. Atchison, it was unanimously

*Resolved*, That in the opinion of this Association, medical colleges ought not to recognize or hold fellowship with any school or its alumni in which irregular medicine is taught as a part of the curriculum.

Question 8. Can college fees be made uniform?

On motion of Prof. Geddings, this question was referred to a committee of five, to report at the meeting of the Association to be held in 1877.

The chair appointed Profs. Geddings, Gross, Angier, E. Curtis and L. Curtis, this committee.

On motion of Prof. Biddle, the following resolution was unanimously adopted:

No degree in medicine should be conferred, under any circumstances, except after an examination in person of the candidate upon all the branches of medicine.

On motion of Prof. Atchison, the thanks of the Association were tendered to the President for the able and impartial manner in which he had discharged the duties of the chair.

On motion of Prof. Yandell, the thanks of the Association were tendered to the Secretary for his efficient services.

On motion of Prof. Larabee, the thanks of the Association were tendered to Jefferson Medical College for the use of the hall and other courtesies.

On motion, the Association adjourned to meet at the call of the President.

J. B. BIDDLE, M. D., President.

LEARTUS CONNOR, M. D., Sec'y.

